# Specific T-Cell Immune Response to SARS-CoV-2 Spike Protein over Time in Naïve and SARS-CoV-2 Previously Infected Subjects Vaccinated with BTN162b2

**DOI:** 10.3390/vaccines10071117

**Published:** 2022-07-13

**Authors:** Natali Vega-Magaña, José Francisco Muñoz-Valle, Marcela Peña-Rodríguez, Oliver Viera-Segura, Ana Laura Pereira-Suárez, Jorge Hernández-Bello, Mariel García-Chagollan

**Affiliations:** 1 Laboratorio de Diagnóstico de Enfermedades Emergentes y Reemergentes, Departamento de Microbiología y Patología, Centro Universitario de Ciencias de la Salud, Universidad de Guadalajara, Guadalajara 44340, Mexico; alejandra.vega@academicos.udg.mx (N.V.-M.); marcee24.p.r@gmail.com (M.P.-R.); o.vierasegura@gmail.com (O.V.-S.); 2 Instituto de Investigación de Ciencias Biomédicas, Centro Universitario de Ciencias de la Salud, Universidad de Guadalajara, Guadalajara 44340, Mexico; biologiamolecular@hotmail.com (J.F.M.-V.); analauraps@hotmail.com (A.L.P.-S.); jorge.hernandezbello@cucs.udg.mx (J.H.-B.)

**Keywords:** T cell, SARS-CoV-2, BTN162b2, spike, vaccines

## Abstract

Due to the COVID-19 pandemic, the rapid development of vaccines against SARS-CoV-2 has been promoted. BNT162b2 is a lipid-nanoparticle mRNA vaccine with 95% efficacy and is the most administered vaccine globally. Nevertheless, little is known about the cellular immune response triggered by vaccination and the immune behavior over time. Therefore, we evaluated the T-cell immune response against the SARS-CoV-2 spike protein and neutralization antibodies (nAbs) in naïve and SARS-CoV-2 previously infected subjects vaccinated with BTN162b2. Methods: Forty-six BTN162b2 vaccinated subjects were included (twenty-six naïve and twenty SARS-CoV-2 previously infected subjects vaccinated with BTN162b2). Blood samples were obtained at basal (before vaccination), 15 days after the first dose, and 15 days after the second dose, to evaluate cellular immune response upon PBMC’s stimulation and cytokine levels. The nAbs were determined one and six months after the second dose. Results: SARS-CoV-2 previously infected subjects vaccinated with BTN162b2 showed the highest proportion of nAbs compared to naïve individuals one month after the second dose. However, women were more prone to lose nAbs percentages over time significantly. Furthermore, a diminished CD154+ IFN-γ+ CD4+ T-cell response was observed after the second BTN162b2 dose in those with previous SARS-CoV-2 infection. In contrast, naïve participants showed an overall increased CD8+ IFN-γ+ TNF-α+ T-cell response to the peptide stimulus. Moreover, a significant reduction in IP-10, IFN-λI, and IL-10 cytokine levels was found in both studied groups. Additionally, the median fluorescence intensity (MFI) levels of IL-6, IFNλ-2/3, IFN-𝛽, and GM-CSF (*p* < 0.05) were significantly reduced over time in the naïve participants. Conclusion: We demonstrate that a previous SARS-CoV-2 infection can also impact cellular T-cell response, nAbs production, and serum cytokine concentration. Therefore, the study of T-cell immune response is essential for vaccination scheme recommendations; future vaccine boost should be carefully addressed as continued stimulation by vaccination might impact the T-cell response.

## 1. Introduction

The Coronavirus Disease 2019 (COVID-19) pandemic, caused by the severe acute respiratory syndrome coronavirus (SARS-CoV-2), has raised the necessity for the rapid development of novel, improved, effective, and safe prophylactic vaccines. Several promising vaccine candidates have emerged, including different platforms such as mRNA, vector-based, and protein-adjuvant vaccines [1,2]. At the time of writing, 472,327,041 cases of COVID-19 have been reported and more than nine billion vaccines doses have been administered around the world with wide vaccination schemes, which could imply that at least half of the global population has developed immunity to SARS-CoV-2, either by vaccination or through natural infection [3].

The BNT162b2 was the first lipid-nanoparticle mRNA vaccine approved by the Food and Drug Administration (FDA) for emergency use; it showed a 95% efficacy in phase II/III trials, demonstrated a reduced laboratory-confirmed infection and viral loads in individuals who were infected, protection against severe COVID-19, as well as a lower hospitalization rate [4,5]. Consequently, BNT162b2 was first approved for emergency use in several countries; hence, it is the most administered vaccine, reporting more than 540 million doses globally [6].

The immunological response triggered by vaccination includes both humoral and cellular immunity. A specific goal for every vaccine design has been to develop a long-lasting immune response; however, since the first vaccines were administered, diverse studies have shown that the immune response, especially the titers of neutralizing antibodies, tends to decrease over time [7,8,9].

Most of the effort has been directed to study the role of antibodies produced after vaccination or natural infection, without considering the cellular immune response, which is also an important indicator of SARS-CoV-2 exposure. Although this remains to be fully demonstrated, the effective response of T cells along with the titers of antibodies might represent key future research to consider for candidate vaccine design [10,11,12]. Circulating SARS-CoV-2-specific T cells have been found in antibody-seronegative convalescent individuals presenting asymptomatic and mild COVID-19, which suggests that, in the absence of antibodies, a robust and broad T-cell response might be sufficient to provide immune protection against SARS-CoV-2 [13]. Moreover, due to the processing of antigens, T-cell responses can face the presence of mutations that otherwise would determine the escape from antibody recognition. Nevertheless, the recognition by T cells of this short sequences derived from the processing of viral antigens, either vaccination or natural infection, associated with HLA class I and II molecules, implies that each individual will be characterized by a distinct print of T cells [14,15,16]; therefore, compared to the antibody analysis, the measurements of SARS-CoV-2-specific T cells are more complex and limited to routinely performed in large numbers for diagnostic purposes. Nonetheless, direct and indirect methods to measure antigen-specific T cells as Peptide-MHC multimer technologies, cytokine release, and activation-induced markers (AIM), such as CD154+ upregulation assay, have been developed to offset this challenge [17,18].

The first study evaluating the T-cell response after the BNT162b2 boost showed a strong IFN-γ+ or IL-2+ response by CD8+ and CD4+ T cells; furthermore, effector-memory phenotype comprised 0.02–2.92% of the total circulating CD8+ T cells, detected for over eight weeks. Important regulators of the immune response are cytokines, in particular, TNF-α, interferons, and IL-1β which are paramount for viral clearance. BNT162b2 can induce the secretion of IFN-γ and IL-2 by CD4+ T cells with a peak on day 29 and decrease around day 43; on the other hand, IL-4 was barely detected [19]. Moreover, cytokines as TNF-α and IFN-γ measured after immunization correlated with higher frequencies of CD8+ and CD4+ T cells [20].

It is important to point out that only a few studies have evaluated the T-cell response efficacy of the anti-SARS-CoV-2 vaccines in patients with previous infection. In fact, most clinical trials have mainly evaluated vaccine efficiency by measuring neutralizing antibodies induction in naïve and SARS-CoV-2 exposed patients after vaccination [20,21]. This research approach might have implications in public health policies as T-cell immunity also plays a critical role in transmission, re-infections, and disease severity; yet, repeatedly immunization either by natural infection [22] or by vaccines has not been fully evaluated and it be could prone to T-cell exhaustion. Due to this, in this paper, we evaluate the impact of a prior history of SARS-CoV-2 infection on the immune response by describing the humoral, cellular, and cytokine response to mRNA vaccination with BNT162b2 compared with those naïve over time.

## 2. Materials and Methods

### 2.1. Study Population

We performed a prospective cohort study, the population consisted of 46 BTN162b2-vaccinated subjects from the Laboratorio de Diagnóstico de Enfermedades Emergentes y Reemergentes (LaDEER) in the Universidad de Guadalajara, Jalisco. Blood samples were obtained in the following time points starting in February 2021—basal, 15 days after the first dose (first BTN162b2 dose), and 15 days after the second dose (second BTN162b2 dose)—to evaluate cellular immune response upon PBMC’s stimulation. Cytokine levels were evaluated in serum samples at the same time point. Additionally, serum samples obtained 1 month (nAbs 1 month) and 6 months after the second dose (nAbs 6 months) were employed for the antibody neutralization assay. Clinical information regarding the vaccine’s side effects and previous COVID-19 infection was also obtained. All participants were negative to SARS-CoV-2 infection by RT-PCR by the time of vaccination.

The study was approved by the local Ethical Committee of the Universidad de Guadalajara (approval number: CI-05621). The study was performed in compliance with the Regulations of the General Health Law on Health Research (1987) and in accordance with the ethical standards noted in the 1964 Declaration of Helsinki and its later amendments or comparable ethical standards.

### 2.2. PBMCs Isolation

Fresh ethylene-diamine-tetra-acetic acid (EDTA) blood samples were used to isolate peripheral blood mononuclear cells (PBMCs) by Ficoll density gradient centrifugation and resuspended RPMI 1640 medium (Gibco, Thermo Scientific, Waltham, MA, USA) with 10% dimethyl sulfoxide (DMSO, Sigma-Aldrich, Burlington, MA, USA), and 20% fetal bovine serum then gradually frozen at −80 °C and cryopreserved in liquid nitrogen gas phase until use. All samples were collected in this manner to assure the future proper simultaneous analysis of the samples that were obtained on different days.

### 2.3. PBMC Culture Conditions and Stimulations

Flow cytometry was implemented to characterize the specific T-cell response against a SARS-CoV-2 lyophilized peptide pool that covered the whole spike protein (Peptivator SARS-CoV-2 Prot-S/S1/S+ Miltenyi Biotec, Bergisch Gladbach, Germany), following the manufacturer’s instructions.

One day before the experiment, PBMCs were thawed, seeded, and incubated overnight at 37 °C and 5% of CO_2_. Subsequently, 10⁶ PBMCs per well were seeded in a 96-well plate in a total volume of 100 µL cell culture medium. Next, cells were stimulated with SARS-CoV-2 PepTivator^®^ SARS-CoV-2 Protein S pool at 0.6 nmol of each peptide/mL, CytoStim was used as a positive control, and DMSO solution at 10% as a negative control. After 2 h of stimulation, Brefeldin A was added and then cells were incubated for an additional 4 h. The cells were washed and fixed according to the protocol. Afterward, cells were stained and incubated for 20 min with the flow cytometry antibody panel provided in the kit, consisting of fluorochrome-labeled monoclonal primary antibodies directed against APC anti-human CD3 (clone REA613), VioBright B515 anti-human CD4 (Clone REA623), VioGreen anti-human CD8 (clone REA734), PE anti-human IFN-γ (clone 45-15), PE-Vio 770 anti-human TNF-α (clone cA2), and APC Vio 770 anti-human CD154 (clone 5C8).

Finally, cells were analyzed using the Attune^®^ NxT flow cytometer (Life Technologies., Carlsbad, CA, USA). The data obtained were analyzed using FlowJo software v10.0 (TreeStar, Inc., Ashland, OR, USA). Lymphocytes were gated using morphological parameters according to their forward scatter (FC) and side scatters (SS) characteristics. Then, using the labeling antibody scheme, further gates were placed around those CD3+ and CD4+ T cells as well as CD3+ and CD8+ T cells. Subsequently, CD154 and IFN-γ expression was evaluated on CD4+ T cell and IFN-γ and TNF-α expression were evaluated on CD8+ T cell. The frequency of antigen-specific T cells was determined by subtracting the frequencies of antigen-specific T cells in unstimulated DMSO controls from peptide-stimulated samples.

### 2.4. Serum Cytokine Profile Presence over the Time

Basal and 15 days after first BTN162b2 dose and second BTN162b2 dose serum samples from 15 SARS-CoV-2 naïve and 8 previously infected patients were analyzed with the LEGENDplex Human Anti-Virus Response Panel (13-plex, Cat No. 740390). The results were analyzed with the LEGENDplex v8 software (BioLegend), and the median fluorescence intensity (MFI) of each analyte was normalized (log10).

### 2.5. Neutralizing Antibodies Determination

Serum patient samples from 1 month and 6 months after second BTN162b2 dose were analyzed for neutralizing antibodies (nAbs). The detection was performed with the cPass™ SARS-CoV-2 Neutralization Antibody Detection Kit (GenScript, Piscataway Township, NJ, USA), according to the manufacturer’s instructions. This test is a blocking Enzyme-Linked Immunosorbent Assay (ELISA) for the semi-quantitative direct detection of total anti-SARS-CoV-2 neutralizing antibodies in human serum. The kit has a 30% signal inhibition cut-off for SARS-CoV-2 neutralizing antibody detection; subsequently, the percent signal inhibition was calculated with the following formula:

% 𝑠𝑖𝑔𝑛𝑎𝑙 𝑖𝑛ℎ𝑖𝑏𝑖𝑡𝑖𝑜𝑛 = (1 − 𝑂𝐷 𝑣𝑎𝑙𝑢𝑒 𝑜𝑓 𝑆𝑎𝑚𝑝𝑙𝑒𝑂𝐷 𝑣𝑎𝑙𝑢𝑒 𝑜𝑓 𝑁𝑒𝑔𝑎𝑡𝑖𝑣𝑒 c𝑜𝑛𝑡𝑟𝑜𝑙) × 100%.

### 2.6. Data Analysis and Statistics

Primary data analysis and statistical analysis were performed using the program IBM SPSS statistics, version 24 for Windows (IBM Corp, Inc., Chicago, IL, USA). A *p*-value < 0.05 was considered statistically significant. The categorical (qualitative) variables were summarized as frequencies and percentages, while continuous (quantitative) variables as mean standard deviation and median and percentiles according to the data distribution evaluated by a Kolmogorov–Smirnov test. A non-parametric Kruskal–Wallis test was used in settings with three or more comparisons and Mann–Whitney U for two. Correlation analyses were performed as non-parametric tests using Spearman statistics. PCA was obtained with the PCA function in the FactoMineR package and visualized with fviz_pca_biplot. We performed a PERMANOVA using the function Adonis from the Vegan R package with Euclidean distance using the grouping of previous and naïve SARS-CoV-2 infection, employing R Studio software v4.1.2 (R Core Team, Vienna, Austria).

## 3. Results

### 3.1. Clinical and Demographic Records of Evaluated Population

Between February and November 2021, 46 people were screened for cytokine profile, anti-SARS-CoV-2 neutralizing antibodies, and T-cell activation response in a follow-up cohort. The participants’ age ranged from 20 to 39, with a mean age of 27.8 ± 4.2. A total of 20 (44.5%) individuals manifested a previous SARS-CoV-2 infection, who were significantly older than the naïve participants (29.83 ± 4.17 vs. 25.89 ± 2.96; *p* < 0.01); in the previous infection group, 12 were male (60.0%). The detailed data are shown in Table 1.

### 3.2. Symptoms Reported after Vaccination

**Figure 1:**
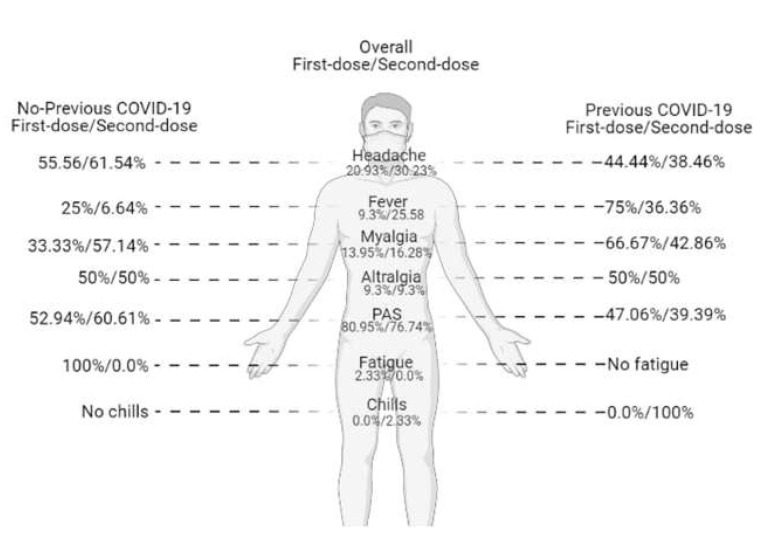
Symptoms reported by the participants after the first and second BTN162b2 doses in the no-previous COVID-19 and previous COVID-19 groups.

Overall, 35 participants (25.93%) reported symptoms after the first BTN162b2 dose, of which 19 were SARS-CoV-2 naïve and 16 had a previous SARS-CoV-2 infection. After both doses, the most reported symptom was pain in the application site (PAS) in both groups, whilst headache was the most reported symptom in the naïve participants and myalgia in the previous SARS-CoV-2 infection group. Fatigue was only reported by one participant from the naïve group during the first dose (Figure 1). Moreover, a risk analysis showed that the population in the previous COVID-19 group had an increased risk to develop symptoms (OR = 0.18; CI: 0.32–0.98; *p* = 0.049).

### 3.3. Neutralizing Antibody Response to BTN162b2 Vaccine

**Figure 2:**
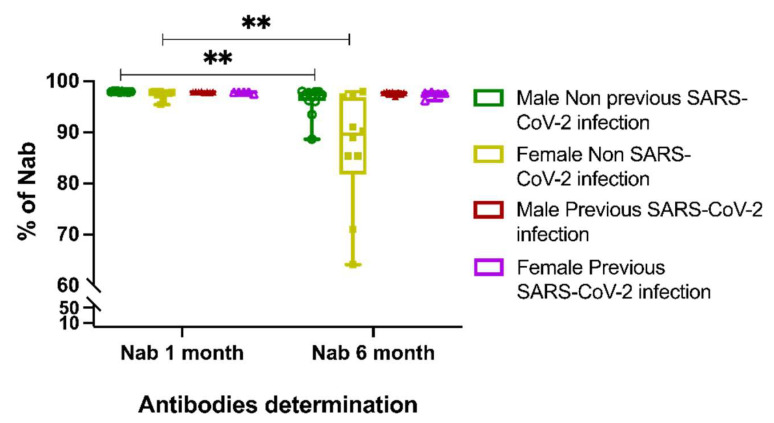
Neutralizing antibodies in the studied population. Female participants, likewise to non-previous COVID-19 groups, showed the greatest loss of neutralizing antibodies in comparison to previous COVID-19 groups and males. Differences between groups were determined by Mann–Whitney U test with an α = <0.05; ** *p* = 0.01–0.001.

After the first month, the BTN162b2-vaccine-induced antibodies in all of the studied individuals had a mean value of 97.73 ± 0.52 neutralizing activity. Previous infection with SARS-CoV-2 showed the highest proportion of nAbs compared to naïve individuals after the first month (97.74 ± 0.44 vs. 97.72 ± 0.58, respectively), but the differences were not significant. Nevertheless, when we performed a determination of the nAbs after six months, the mean was 93.93 ± 7.69, representing a considerable decay (*p* < 0.001) in contrast to the first month, especially in those SARS-CoV-2 naïve, who tended to have a higher rate of nAbs loss (91.37 ± 9.26 vs. 97.56 ± 0.47; *p* = 0.01). Additionally, in line with the latter, a sex segregation evaluation was performed; women were more prone to significantly lose nAbs percentages over time (from 97.37 ± 0.84 to 86.79 ± 11.23; *p* = 0.004). In contrast, males in the first month showed a mean of 97.92 ± 0.09, and at month six, they decreased to 96.14 ± 2.81 (*p* = 0.003). This trend was not observed in the participants who reported a previous COVID-19 infection (males at month one 97.87 ± 0.09 vs. at month six 97.65 ± 0.31, *p* = 0.75; females at month one 97.61 ± 0.60 vs. at month six 92.69 ± 0.62; *p* = 0.31) (Figure 2).

### 3.4. T-Cell Response against the Peptide Pool of SARS-CoV-2 Spike Protein

To characterize the human T-cell response against SARS-CoV-2, we stimulated PBMC with a peptide-pool of the SARS-CoV-2 spike protein. To achieve this, an ex vivo stimulation of peripheral blood mononuclear cells (PBMCs) for 7 h with overlapping peptide pools was performed.

In this paper, CD4+ and CD8+ T-cell populations reactive to SARS-CoV-2 were detected by the expression of CD154+ (CD40L+)/IFN-γ+, and IFN-γ+/TNF-α+, respectively, where IFN-γ is an important marker of an induced anti-viral immune response [23]. CD154, also called CD40L, is expressed by all-activated conventional CD4+ T-cell subsets and serves as a marker to analyze antigen-specific T cells, due to its transient expression on the surface of CD4+ T cells when activated via T-cell receptor engagement, which is highly specific to evaluate a specific T-cell activation [19].

**Figure 3:**
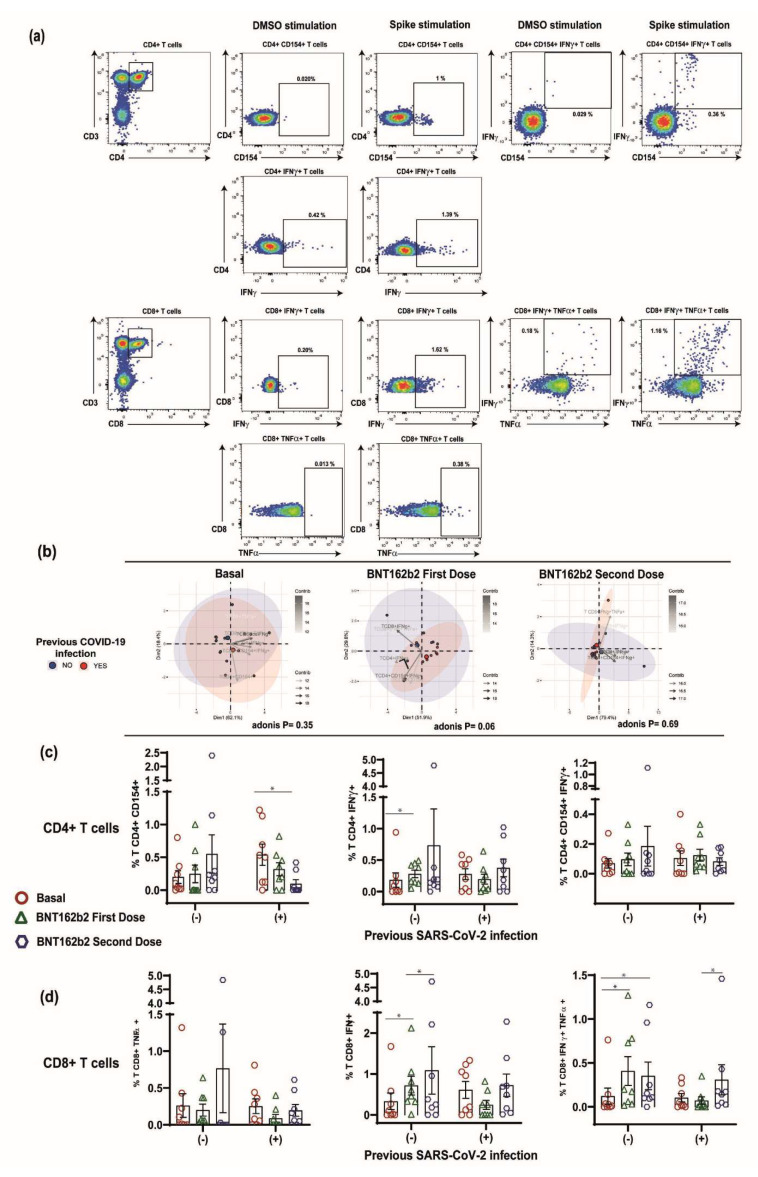
Stimulation of PBMC with a peptide pool of the SARS-CoV-2 spike protein. (**a**) Representative example shows the gated CD4+ and CD8+ T-cell populations. Subsequently, we analyzed the CD4+ CD154+ T cells, CD4+ IFN-γ+ T cells, and CD4+ CD154+ IFN-γ+ T cells as well as CD8+ IFN-γ+ T cells, CD8+ TNF-α+ T cells, and CD8+ IFN-γ+ TNF-α+ T cells. Upon peptide pool stimulation, the results were compared to the negative control (DMSO). (**b**) PCA biplot showing PCA analysis of T-cell response compared over time: previous SARS-CoV-2 infection (red) vs. naïve participants (blue); arrows are drawn based on the percentage contribution of variables to each component. (**c**,**d**) Individual values of CD4+ and CD8+ T-cell populations reactive to SARS-CoV-2 are analyzed following timepoints: basal, 15 days after the first BTN162b2 dose, and 15 days after the second BTN162b2 dose. Differences between groups were determined by Mann–Whitney U test with an α = < 0.05; * *p* = 0.05–0.01.

In order to evaluate the role of T-cell response in participants with a prior history of SARS-CoV-2 infection, we included eight naïve and eight SARS-CoV-2 previously infected participants in the following time points: basal, 15 days after the first BTN162b2 dose, and 15 days after the second BTN162b2 dose. Upon peptide pool stimulation, the results from the participants’ PBMCs were compared to the negative control (DMSO), and the representative results are shown in Figure 3a.

Additionally, we constructed a PCA biplot to find potential clusters and observe trends and patterns of the T-cell response in both groups; it is important to mention that this biplot can also show if some of the variables are negatively or positively correlated. In this sense, our data showed a variation according to a previous SARS-CoV-2 infection over time; in the basal time point and after the first BTN162b2 dose, the samples (represented by colored dots) formed different clusters according to a previous infection. However, after the second dose, both groups formed one cluster (Adonis, *p* = 0.693) (Figure 3b). Regarding the frequencies of reactive cells at the basal time point, the previous SARS-CoV-2 group showed a CD154+ CD4+ (0.54 ± 0.46 vs. 0.2 ± 0.27; *p* = 0.10), IFN-γ+ CD4+ (0.28 ± 0.24 vs. 0.18 ± 0.32; *p* = 0.34), and CD154+ IFN-γ+ CD4+ (0.1 ± 0.14 vs. 0.07 ± 0.09; *p* = 0.92) T-cell increased response compared to the naïve group; however, this difference was not significant. After the first BTN162b2 dose, the previous SARS-CoV-2 group showed a CD154+ CD4+ (0.32 ± 0.28 vs. 0.25 ± 0.38; *p* = 0.45), IFN-γ+ CD4+ (0.2 ± 0.22 vs. 0.28 ± 0.17; *p* = 0.25), and CD154+ IFN-γ+ CD4+ (0.12 ± 0.11 vs. 0.1 ± 0.12; *p* = 0.34) T-cell increased response compared to the naïve group, but with no significant differences (Figure 3c). Furthermore, a clearly diminished CD154+ CD4+ (0.1 ± 0.17 vs. 0.55 ± 0.83; *p* = 0.15), IFN-γ+ CD4+ (0.37 ± 0.4 vs. 0.73 ± 1.64; *p* = 0.83), and CD154+ IFN-γ+ CD4+ (0.08 ± 0.07 vs. 0.19 ± 0.38; *p* = 0.56) T-cell response was observed after the second BTN162b2 dose in the patients with previous SARS-CoV-2 infection compared to the non-previous infection group. Interestingly, when considering only the previous SARS-CoV-2 infection group, T-cell reaction had a clear diminished response over time in the basal and second dose groups, 0.54 ± 0.46 vs. 0.1 ± 0.17 (*p* = 0.015), respectively. Moreover, no differences between sexes were observed on CD4+ and CD8+ T-cell activation, nor over time (data not shown).

On the other hand, the CD8+ evaluation was performed based on CD8+ TNFα+, CD8+ IFNγ+, and CD8+ TNFα+ IFNγ+ expression. Within CD8+ T-cell populations, IFN-γ and TNF-α are cytolytic effector molecules [23,24]. In this sense, overall, CD8+ IFNγ+ T cells had a mirrored response compared to CD4+ IFNγ+ T cells in both of the studied populations. In contrast with our findings in the CD4+ population, we did not observe a decreased reactivity to the stimulation in the CD8+ TNFα+ and CD8+ TNFα+ IFNγ+ T cells over time. Moreover, when comparing with a previous SARS-CoV-2 infection, the naïve participants showed an overall increased response to the peptide stimulus, in specific, the major response was observed after the second BTN162b2 dose. Interestingly, in the previous SARS-CoV-2 infection group, a notorious decrease in the CD8+ response markers after the first BTN162b2 dose was observed, which was partially recovered after the second BTN162b2 dose, but with a lower reactivity than in the naïve group (Figure 3d). As for CD8+, similar to CD4+ T cells, no differences among sex were observed on CD4+ and CD8+ T-cell activation.

### 3.5. Serum Cytokine Profile among the BTN162b2-Vaccinated Participants

**Figure 4:**
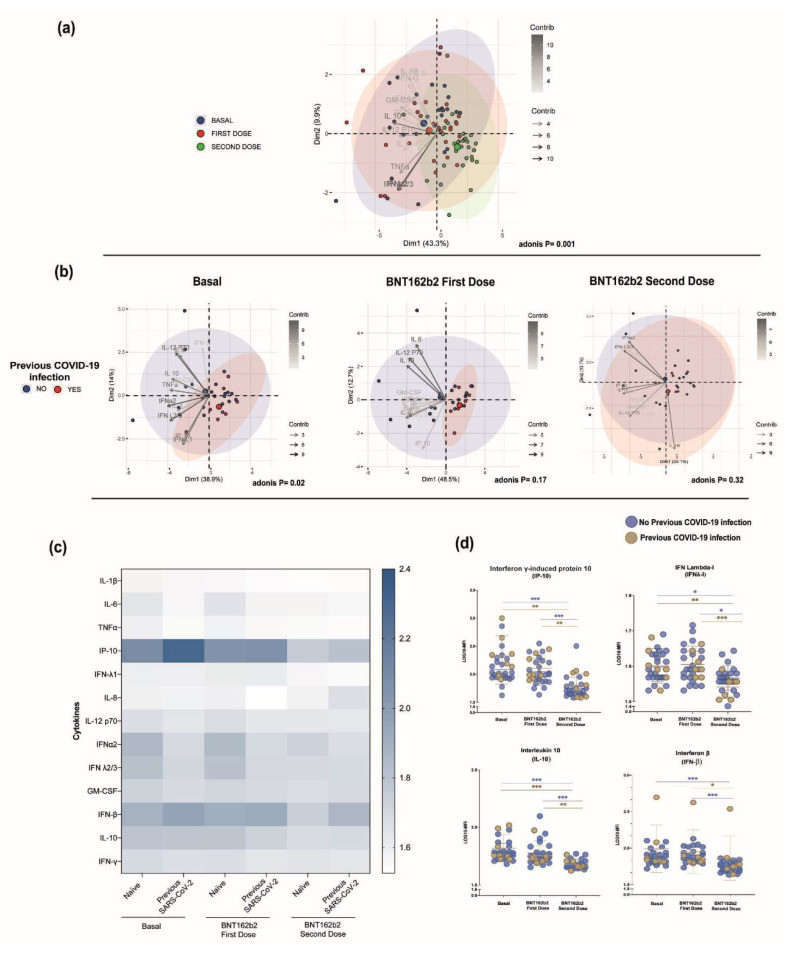
(**a**) PCA biplot showing the PCA analysis of serum cytokine profile compared over time and (**b**) a previous SARS-CoV-2 infection vs. naïve participants; plots were generated according to each time point. Principal component scores for components 1 (PC1) and 2 (PC2) were plotted for each group and time point. Samples were then colored by group; previous SARS-CoV-2 infection (red) and naive (blue) illustrate the sample clustering. Arrows are drawn based on the percentage contribution of variables to each component. (**c**) Heatmap of the cytokine MFI log values compared over time and a previous SARS-CoV-2 infection. (**d**) Individual cytokine values of the representative cytokines from the analyzed participants. Differences between groups were determined by Mann–Whitney U test with an α ≤ 0.05; * *p* = 0.05–0.01, ** *p* = 0.01–0.001, *** *p* < 0.001.

A panel of 13 cytokines related to viral response was measured in naïve and SARS-CoV-2 infected participants; the results were compared among these groups and stratified over time. A correlation clustering analysis of the cytokines according to the BTN162b2 vaccine doses was constructed and we observed a notable distinction among the groups; moreover, the clustering was more evident after the second BTN162b2 dose (Figure 4a). This was confirmed through the Adonis analysis (*p* < 0.001). Consequently, in order to evaluate if a previous SARS-CoV-2 infection influenced the clustering, we analyzed each time point according to this classification through a PCA biplot. At the basal timepoint (*p* = 0.02), the segregation of the samples was more evident than in the first BTN162b2 dose (*p* = 0.17). Furthermore, after the second BTN162b2 dose, this segregation was lost and both groups clustered together (*p* = 0.33) (Figure 4b). No differences were observed when the data were grouped according to sex over time (Adonis = 0.09).

Due to the observed PCA results, we visualized the samples in a heatmap to evaluate the concentration differences between the naïve and previous SARS-CoV-2 infection groups and over time (Figure 4c,d). A significant reduction in IP-10, IFN-λI, and IL-10 cytokine levels were found in both of the studied groups when comparing the basal vs. second BTN162b2 dose (*p* < 0.05) and first BTN162b2 dose vs. second BTN162b2 dose (*p* < 0.05) time points. Notably, most of the differences were found in the group with no previous COVID-19 infection. For instance, when comparing the basal vs. second dose and first dose vs. second dose time points, the MFI levels of IL-6, IFNλ-2/3, IFN𝛽, and GM-CSF (*p* < 0.05) were significantly reduced over time in the naïve participants. Meanwhile, IFN-γ levels constantly decreased from basal to the second dose (*p* < 0.05), and IL-1𝛽, IFNα2, and TNF-α levels were significantly reduced only from basal to the second dose time point (*p* < 0.05). In respect to the participants with previous SARS-CoV-2 infection, a significant reduction was found in IL-1𝛽 levels from basal to the second BTN162b2 dose time point, as well as in the IFN-𝛽 levels (*p* < 0.05) from the first BTN162b2 dose to the second BTN162b2 dose.

On the other hand, when compared with a previous SARS-CoV-2 infection, naïve participants showed higher levels (*p* < 0.05) in basal IFN-α2. Likewise, IFN-α2 levels were higher in the first dose and this was significant in the second dose time point (*p* = 0.05).

## 4. Discussion

Former studies based on the efficacy and safety of the new generation vaccines against COVID-19 have demonstrated the importance of evaluating cellular and humoral immune responses. Novel vaccine technologies, such as BNT162b2, stimulate a broad range of immune responses [6,25], but some of those mechanisms remain fully uncharacterized. In this paper, we applied the technology of a spike-SARS-CoV-2 peptide pool to study T cells’ immunological response, peripheral cytokine profile, and neutralizing antibodies over time after immunization with an mRNA-based vaccine, comparing the response among naïve and SARS-CoV-2 previously infected participants. Our analysis showed decay in neutralizing antibodies after six months of BNT162b2 boost, which was pronounced in females from the naïve group. Moreover, serum cytokine profile became homogeneous after the BNT162b2 boost, while a heterogeneous T-cell response over time in CD4+ and CD8+ was observed in both of the studied groups.

Studies evaluating the influence of sex in the maintenance of neutralizing antibodies over time have shown disparities. Our study showed that female participants are prone to a rapid decay of nAbs after six months, regardless of the previous infection with SARS-CoV-2; however, other studies evaluating nAbs at six months after the first dose did not find differences among sex, although a visible loss was observed [20]. Notably, another study showed that, after six months of the second BNT162b2 dose, the nAbs titers were lower in men than among women, contrary to the results obtained in this paper. Regarding the effect of the previous SARS-CoV-2 natural infection, likewise to other studies, the vaccine boosts the humoral response, extending the time for high levels of nAbs after six months. In convalescent individuals, males demonstrated robust antibody durability three months after vaccination compared with women [26]. This disparity in the sex-dependent antibody levels can be due to different factors that have been hypothesized, such as a combination of sex hormone effect, micro-RNA levels, immune gene expression related to the X chromosome, and genetic polymorphism of immunological proteins, such as IL-6 or CTLA-4 [27,28,29].

Mature CD8+ and CD4+ T-cell populations play a significant role in the defense against viral clearance. CD8+ cytotoxic cells are capable of secreting an array of molecules, such as perforin, granzymes, and cytokines, such as IFN- γ, to eradicate infected cells from the host, while CD4+ T-helper cells can assist other cells of the immune system by enhancing the ability of cytotoxic T cells and B cells to clear pathogens [30]. The cellular immune response is essential since SARS-CoV-2 variants have demonstrated to affect recognition by neutralizing antibodies. However, the T-cell response is less susceptible to viral mutations and appears to be longer-lasting in the human body than antibodies; therefore, the understanding of how different vaccine platforms induce a long-term T-cell response has become crucial for the development and improvement of vaccines [15,31].

When vaccine efficacy was evaluated in clinical trials, they excluded individuals with a prior diagnosis of COVID-19 [6,25]; therefore, little is known about how mRNA vaccine responses behave following a previous natural infection. In the present study, it was evaluated the T-cell response on participants vaccinated with BTN161b2 in a cohort following time points: basal, 15 days after the first BTN162b2 dose, and 15 days after the second BTN162b2 dose; in addition, participants with a prior history of SARS-CoV-2 infection were included and compared with naïve participants for SARS-CoV-2 infection. In our correlation clustering analysis over time with either CD4+ and CD8+ specific T-cell populations, although no statistical differences were observed, it was clearly seen how, at the basal point and after the first BTN162b2 dose, participants who already experienced a SARS-CoV-2 infection formed a separate cluster, pointing to a different behavior compared to naïve participants. This result is consistent with data previously shown, where either neutralizing antibodies and T-cell response evaluated by T-cell enzyme-linked immunospot (ELISpot) assays after a single dose of BTN162b2 mRNA vaccine were more robust in individuals with previous infection compared to the very little response of infection-naive individuals. Interestingly, after the second dose, specific T cells from both naïve participants and those with a previous COVID-19 infection formed one cluster and they behaved more homogeneously. However, when the T-cell response was evaluated by previous SARS-CoV-2 infection, a clearly diminished response in CD4+ and CD8+ T cells was observed after the second BTN162b2 dose between the naïve and SARS-CoV-2 previously infected participants; this same behavior of the T cells was observed in a recent study in which a poorly T-cell response was reported in the vaccinated population with the previous infection, denominated by the author as a muted T cells [23].

In particular, CD4+ CD154+ T-cell response in the previous SARS-CoV-2 infection group was notoriously reduced over time, even when compared with its naïve counterpart. This contraction in the response has previously been associated with a persistent stimulation, which in turn may induce T-cell exhaustion, leading to loss of cytokine production capability and reduced functionality [22,24]. Whilst exhaustion markers were not measured in this study, it has been demonstrated that CD40L levels are reduced with antigenic overstimulation [32].

With respect to the cytokine serum profile, the constant in both groups was a significant reduction in the serum cytokine level over time; nevertheless, most of the differences were found in the naïve group with no effect observed from basal to the first BTN162b2 dose. This could have been due to the timing of sample recovering; previous studies have demonstrated higher serum cytokine levels within the first days of first and second vaccination doses [19]. As to the reduction observed in the cytokine levels, this could be explained by the constant antigenic stimuli by the BTN162b2 vaccine since it can impair the immune response [32,33].

It is important to mention that the time point when the serum cytokines were measured is a limiting factor of this study. Peak serum cytokine levels after the BNT162b2 vaccine have been reported 6 h after the first dose in mice [34], and 3 days after in humans [35]. However, some patients, also called “slow responders”, reach their maximum cytokine concentrations 20/24 days after the first dose of the vaccine. This variation has been reported to be influenced by sex, hypertension, abdominal obesity, and smoking habit [36]. In this paper, we demonstrated that a previous SARS-CoV-2 infection can also impact cellular T-cell responses, nAbs production, and serum cytokine concentration.

In the quest for answers to the effect of the BTN162b2 vaccine on the cytokine profile, we found significant type I IFN modifications and higher IFNα-2 levels only seen after the first dose in the previous SARS-CoV-2 infection group, which might be related with a better cellular immune response as well as nAbs generation. This relationship has been described by Chunfeng Li et al. (2022) who conclude that the MDA5–IFNAR1 signaling pathway is critical for the CD8+ T-cell response induced by BNT162b2 [34]. This is in line with our results where IFN-α2 is the major contributing variable in the PCA analysis (11%; data not shown). It is important to mention that, even though type I IFNs are extremely important, it is recommended that naïve CD8+ T cells are primed first by the TCR–MHC-I signal and later by the IFN-I, to properly mount CD8+ T-cell activation and proliferation instead of apoptosis [37].

## 5. Conclusions

In this study, we show that CD154, which is an important marker related to T-cell activation after antigen stimulation, is altered depending on a previous SARS-CoV infection. Specifically, we observed that the previous SARS-CoV-2 infected group presented a diminished frequency of CD4+ CD154+ T-cell populations after the second dose of the BNT162b2 vaccine compared to the naïve participants. Therefore, it might be recommended that public health measures in future vaccine boosts should be carefully addressed as continued stimulation by vaccination might impact T-cell activation, although further studies in this field are necessary.

## Figures and Tables

**Table 1:** Demographic, clinical, and antibody records.

	Total n (%)	No Previous COVID-19 Mean ± SD	Previous COVID-19 Mean ± SD
n (%)	46 (100%)	26 (55.5%)	20 (44.5%)
Age (mean ± SD)	27.8 ± 4.2	25.89 ± 2.96	29.83 ± 4.17
Sex			
Male	25 (54.3%)	13 (50.00%)	12 (60.00%)
Female	21 (45.7%)	13 (50.0%)	8 (40.00%)
BMI	24.04 ± 8	26.26 ± 4.73	21.10 ± 10.4

SD: standard deviation.

## Data Availability

The data presented in this study are available upon request from the corresponding author (M.G.-C.). The data are not publicly available due to privacy concerns.
